# A supervised learning approach for taxonomic classification of core-photosystem-II genes and transcripts in the marine environment

**DOI:** 10.1186/1471-2164-10-229

**Published:** 2009-05-16

**Authors:** Shani Tzahor, Dikla Man-Aharonovich, Benjamin C Kirkup, Tali Yogev, Ilana Berman-Frank, Martin F Polz, Oded Béjà, Yael Mandel-Gutfreund

**Affiliations:** 1 Faculty of Biology, Technion – Israel Institute of Technology, Haifa 32000, Israel; 2 Inter-Departmental Program for Biotechnology, Technion – Israel Institute of Technology, Haifa 32000, Israel; 3 Department of Civil and Environmental Engineering, Massachusetts Institute of Technology, Cambridge, MA 02139, USA; 4 Faculty of Life Sciences, Bar-Ilan University, Ramat Gan 52900, Israel

## Abstract

**Background:**

Cyanobacteria of the genera *Synechococcus *and *Prochlorococcus *play a key role in marine photosynthesis, which contributes to the global carbon cycle and to the world oxygen supply. Recently, genes encoding the photosystem II reaction center (*psbA *and *psbD*) were found in cyanophage genomes. This phenomenon suggested that the horizontal transfer of these genes may be involved in increasing phage fitness. To date, a very small percentage of marine bacteria and phages has been cultured. Thus, mapping genomic data extracted directly from the environment to its taxonomic origin is necessary for a better understanding of phage-host relationships and dynamics.

**Results:**

To achieve an accurate and rapid taxonomic classification, we employed a computational approach combining a multi-class Support Vector Machine (SVM) with a codon usage position specific scoring matrix (cuPSSM). Our method has been applied successfully to classify core-photosystem-II gene fragments, including partial sequences coming directly from the ocean, to seven different taxonomic classes. Applying the method on a large set of DNA and RNA *psbA *clones from the Mediterranean Sea, we studied the distribution of cyanobacterial *psbA *genes and transcripts in their natural environment. Using our approach, we were able to simultaneously examine taxonomic and ecological distributions in the marine environment.

**Conclusion:**

The ability to accurately classify the origin of individual genes and transcripts coming directly from the environment is of great importance in studying marine ecology. The classification method presented in this paper could be applied further to classify other genes amplified from the environment, for which training data is available.

## Background

Marine cyanobacteria *Prochlorococcus *and *Synechococcus *constitute the main prokaryotic fraction of oceanic phytoplankton [1-5]. Their photosynthetic membrane contains two reaction centers, of which photosystem II (PSII) mediates the transfer of electrons and protons from water, the terminal electron donor, to the plastoquinone pool. The D1 and D2 proteins form the reaction center of PSII, which binds the primary electron donors and acceptors. The genes coding for the D1 and D2 proteins, *psbA *and *psbD*, were found recently in the genomes of cyanophages from the *Myoviridae *and *Podoviridae *families [6-10] and were readily detected in recent marine metagenomics projects [11-14]

Recently, a possible exchange and reshuffling of *psbA *genes between *Synechococcus *and *Prochlorococcus *bacteria by horizontal gene transfer (HGT) via phage intermediates was proposed [15,16]. Furthermore, observations based on data extracted from the Global Ocean Sampling (GOS) expedition [11] suggested that the phage genes undergo an independent selection for distinct D1 proteins [12]. The D1 PEST-like domain (implicated as the site of initial cleavage in the D1 protein that initiates protein turnover [17]) in the loop between helices D and E was identified as the target for this viral selection, having specific viral motifs. The D1 protein has a rapid turnover and must be replaced continuously in order to enable the sustained functioning of PSII (due to D1 sensitivity to photodamage [18]). The fact that viruses invest in modifying the D1 proteins led to the hypothesis that an adaptive role for this function is involved, facilitating adaptation to harsh light conditions or modifying the D1 role for their selfish benefits (a more stable D1 that is functional only for the short time of infection). In addition, viral specific *psbA *transcripts were detected directly in one marine sample, implicating that these viral photosynthetic proteins are expressed in the marine environment [12]. These novel observations requires further investigation with much more samples taken at different locations and times, with both genomic DNA and RNA transcripts sampled.

Based on early genomic studies, it was concluded that different species have a unique sequence composition, which has been named 'genomic signature' [19]. The existence of specific genomic compositions has been demonstrated for short oligonucleotides, such as dinucleotides, trinucleotides and tetranucleotides [20-23]. Based on differences in the genomic composition of bacterial and eukaryotic genomes, supervised and unsupervised classification methods have been developed, such as the Naïve Bayesian classifier [24] and the neural network Self-Organizing Maps (SOM) [25]. Characterization and classification of species based on the genomic composition of longer oligonucleotides, such as the Chaos Game Representation (CGR) [26], has also been demonstrated. Several of these approaches have been developed and applied to specifically identify HGT events based on genomic signatures [19,24-27].

The number of unknown taxonomic origin fragments coming directly from the environment has grown rapidly in the past few years [11,14,28-30]. Hence, tools that couple such fragments to their taxonomic origin are extremely valuable for metagenomics research [31-34]. To better describe and understand the phenomenon of marine viral 'photosynthesis' and to further study the HGT of core photosystem-II genes between phages and bacteria, we studied *psbA *genes and transcripts obtained directly from the environment. For rapid classification, we employed a powerful approach that combines genomic composition and position-specific codon usage to identify the specific taxonomic origin of the fragments (100–729 bp). Overall, for binary classification our method achieved very high accuracy when tested on annotated *psbA *fragments from the GOS (86–98% overall accuracy depending on fragment length). To further verify the accuracy of the multi-group classification method and its applicability to similar classification problems, we tested it on an independent dataset of viral *psbA *sequences from a recent study by Chenard and Suttle (2008) [35] that included freshwater samples, as well as on annotated *psbD *gene fragments from the GOS. Overall, our results were highly consistent with original annotations (92% accuracy). Finally, we applied the method to a large set of *psbA *clones, including DNA and transcribed RNA sequences from the Mediterranean Sea. We found that the distribution of bacterial taxa (hosts) was highly correlated when examining the genes versus the transcripts, whereas no such correlation was observed for the phages.

## Results and Discussion

In order to shed light on the diversity and dynamics of marine cyanobacteria and their phages, we examined the spatial and seasonal distribution of *psbA *genes and transcripts in the eastern Mediterranean Sea. We sampled two locations: a coastal station (Tb04) and a pelagic station (Tb01) during the years 2006 and 2007. *PsbA *genes and transcripts from mixed picoplankton assemblages were PCR amplified directly from DNA or RNA extracts using newly designed *psbA *primers [4]. Although these primers are general and amplify *psbA *from picoeukarya, cyanobacteria and cyanophages, they are not biased against high GC environmental *Synechococcus psbA*s (data not shown). It should be noted that the amplified PCR products reported in this study are unique only to this primer set.

A total of 1,205 randomly picked clones containing *psbA *inserts (median length 699, ranging between 414 and 702) were sequenced; 618 were DNA-derived clones and 587 were retrieved from RNA. As a first step in sorting phage and cyanobacterial *psbA *genes and transcripts, picoeukaryal *psbA*s were removed based on phylogenetic trees and top BLAST hits. Interestingly, while almost undetectable in the DNA extracts, eukaryal *psbA *transcripts dominated in several stations and could reach up to 88% of the total *psbA *RNA (data not shown). This observation is in good agreement with longstanding observations that different samples numerically dominated by cyanobacteria (cell abundance) are in fact dominated (activity) by picoeukaryotes [36].

### Classifying psbA fragments based on genomic composition using SVM

The D1 protein sequence is very well conserved amongst bacteria and viruses except for two regions [12]. The first variable region lies within the PEST-like domain located in the loop between transmembrane helices D and E. The second variable region is the viral D1, which differs from the cyanobacterial sequences at the end of transmembrane helix E and precedes the C-terminal residues of mature cyanobacterial D1. These regions possess different sequence motifs that distinguish between the phages and their hosts [12]. However, since the vast majority of the protein sequence is highly conserved, taxonomic classifications that rely solely on these motifs are prone to error. Based on the hypothesis that *psbA *has undergone horizontal transfer events between the bacteria to the phages, its sequence composition may reflect two major processes acting at both the protein (functional) and the DNA level. At the protein level, adaptive changes could make viral D1 proteins less susceptible to photo-damage and could influence its turnover. Additionally, transferred genes are prone to amelioration processes that cause them to reflect the DNA composition of the received genome over time [37].

Previous studies have attempted to distinguish bacterial from viral psbA genes based on GC content [15,16]. In *Synechococcus*, the GC composition differs significantly between the host and its phage: for example, *Synechococcus *phage *psbA *sequences have a noticeably lower percentage of GC [15]. Such differences in GC content were not observed in *Prochlorococcus *phages and their host *psbA *sequences, therefore these differences are not sufficient to unambiguously assign sequences to the phage or the host. To uniquely identify the *psbA *origin based on nucleotide composition information, we chose to employ a machine learning technique called Support Vector Machine (SVM) [38]. SVM has been shown to be a very successful classification tool for biological problems [39,40] including phylogenetic characterization [27,31]. In this study, the input vectors for the SVM were combined from nucleotide composition information (mono-, di-, tri-, tetra- nucleotide frequency) of each *psbA *fragment.

**Figure 1:**
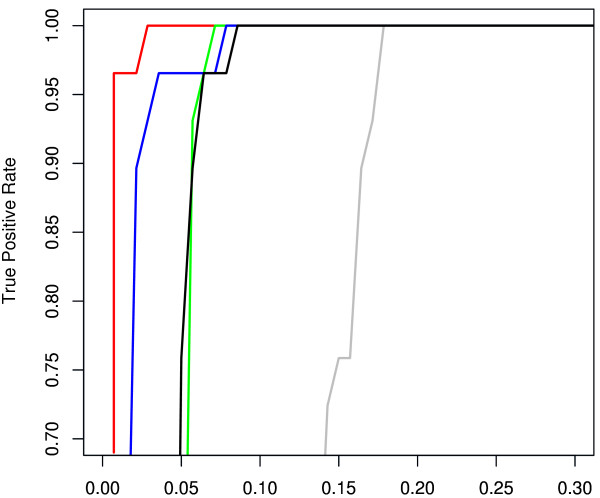
**ROC plot demonstrating the SVM performance for classifying host vs. phage *psb*A trusted sequences from GOS **[11]**based on genomic composition: mononucleotides (gray line), dinucleotides (green line), trinucleotides (blue line), tetranucleotides (red lines), all oligonucleotides (black lines)**. AUC (Area Under Curve) values are given in Table 1. As illustrated, best performance was achieved when including only the tetranucleotide composition.

Our SVM training set was derived from 11 and 15 cultured *Prochlorococcus *host and phage *psbA *sequences, respectively. In addition, 28 and 16 *Synechococcus *host and phage sequences were extracted from cultured data [See Additional file 1]. As a first step, we tested the method on an independent set of 280 *psbA *fragments from the GOS dataset [11]. Applying the SVM classifier based on nucleotide composition, each fragment in the testing set was labeled as *Prochlorococcus *bacteria, *Prochlorococcus-*like phage *Synechococcus *bacteria and *Synechococcus-*like phage. To evaluate the accuracy (sensitivity and specificity) of the SVM, we compared our results to the original classification derived from phylogenetic analysis, based on DNA trees[15], and neighboring genes [12] [See Additional file 2]. Overall, when combining the frequency of mono-, di-, tri- and tetranucleotides in our feature vector, we correctly classified 93% and 100% of the *Prochlorococcus *and *Synechococcus *sequences, respectively. Detailed results for *Prochlorococcus *classifications are given in Table 1 and illustrated in Figure 1.

**Table 1:** SVM prediction results for *Prochlorococcus *classification.

**Feature Vector**	**Test ROC**	**Sensitivity**	**Specificity**	**Accuracy**
Mononucleotide	0.864	0	91.43	75.74

Dinucleotide	0.959	82.76	95	92.9

Trinucleotide	0.989	96.55	95.71	95.86

Tetranucleotide	0.999	96.55	100	99.4

All oligonucleotides	0.955	82.76	95	92.9

Given the differences in GC content between *Synechoccoccus *and *Synechoccoccus*-like phages, a high performance of the classification was expected. Nevertheless, the extremely high prediction accuracy for classifying *Prochlorococcus *vs. *Prochlorococcus*-like phages was not anticipated. In order to better understand which of the oligonucleotide features contribute to the classification, we performed a simple feature selection analysis, each time training and testing the SVM on a different set of oligonucleotide composition (i.e. dinucleotides vs trinucleotides). The results of the SVM tests are illustrated in Figure 1 (full details are given in Table 1). As expected, the mononucleotide frequencies were least informative, achieving an overall accuracy of 75% with 0% sensitivity due to no true positive results (Table 1). As previously demonstrated for genomic sequences [41], the tetranucleotide frequencies differentiated best between host and phage *psbA *sequences with a prediction accuracy of 97%. When including all oligonucleotide frequencies in the feature vectors (Figure 1, blue line), classification performance decreased, giving very similar results as when including only dinucleotide frequencies (Figure 1, black line). Overall, the SVM results tested on *psbA *gene fragments from the GOS data were very promising. As most of the *psbA *fragments from GOS were of full length (average length 679 ± 66 bp), we were interested to test how well the SVM performed on shorter fragments anticipated from metagenomics studies. To examine this, we tested the SVM including only the tetranucleotide frequencies on randomly selected short *psbA *fragments from the GOS data of length 300, 200 and 100 bp. As shown in Table 2, the average prediction accuracy for classifying *Prochlorococcus *vs. *Prochlorococcus*-like phages was extremely high for short fragments of length 300 bp and 200 bp. A considerable reduction in performance was observed for the short sequences of length 100 bp. It is important to note that SVM is a supervised approach that requires the existence of trustworthy data for training. Thus, this method could not be applicable for the taxonomic classification of any random short sequences that are not homologues to the training dataset. This result is consistent with a recent study for classifying short fragments based on Blast hits [42]. Nevertheless, given a set of training data for a specific gene, use of the SVM is possible for automatically defining whether a random short sequence fits the gene model and subsequently classifying it as a viral or bacterial origin.

**Table 2:** SVM and cuPSSM results for short fragments.

**Length**	**Classifier**	Sensitivity*	Specificity*	Accuracy*
300 bp	SVM (tetra)	97.33 ± 1.2	96.55 ± 3	97.5 ± 1.2

	cuPSSM	98.42 ± 0.64	98.39 ± 1.7	98.44 ± 0.7

200 bp	SVM(tetra)	93.51 ± 1.7	90.31 ± 5.7	94.17 ± 1.7

	cuPSSM	98.34 ± 1.7	98.37 ± 1.7	97.32 ± 1

100 bp	SVM(tetra)	85.21 ± 2.6	80.03 ± 7.4	86.29 ± 2.8

	cuPSSM	91.92 ± 1.87	95.27 ± 3.7	91.22 ± 2.1

* The analysis of randomly chosen fragments for sequences of lengths 300, 200 and 100 bp was repeated 30, 50 and 100 times, respectively.

### Classifying psbA fragments based on codon usage using cuPSSM

Another common technique for genome classification is based on codon usage frequencies [43]. To test whether differences in codon usage at each position of the D1 proteins can be applied to the taxonomic classification of short fragments of highly conserved genes, we constructed a codon usage Position Specific Scoring Matrix (cuPSSM) for each set of training data (see Methods section). For comparison, we derived a standard PSSM based solely on amino acid frequency. In a standard PSSM, each column in the matrix corresponds to a single residue in the aligned D1 protein, and each line represents the frequency of an amino acid at each position. Notably, we found that classification based on codon usage information was significantly more accurate (99% accuracy; 96.6% and 100% sensitivity and specificity, respectively) than classification based on amino acid information alone (89% accuracy; 96.6% and 87.9% sensitivity and specificity, respectively). Overall, the results obtained using the cuPSSM for full fragments were comparable to the SVM results based on overall nucleotide composition when ignoring the position information. In order to examine whether the prediction depends on variable regions, such as the PEST-like domain and the end of transmembrane helix E in the C-terminal, we reproduced the same tests excluding these regions. While the PEST-like domain was found to be obligatory for classifying D1 proteins at the amino acid level, it was not required for classification when depending on either the codon usage or the nucleotide signature of the gene. Further, as shown in Table 2, when applying the cuPSSM to randomly chosen partial sequences, a very high accuracy was achieved even for short sequences of 100 bp. These results reinforce that both the SVM and the cuPSSM methods are applicable for classifying the viral or bacterial origin of short *psbA *fragments. However, the main disadvantage of these approaches is that they can only be applied to genes for which training data are available; they cannot be used for the classification of any random fragment obtained from metagenomics studies. In comparison to SVM, the cuPSSM approach depends greatly on the quality of the alignment of the query sequence to the training data and thus can only be applicable for highly conserved genes.

### A combined approach for fragment classification

**Figure 2:**
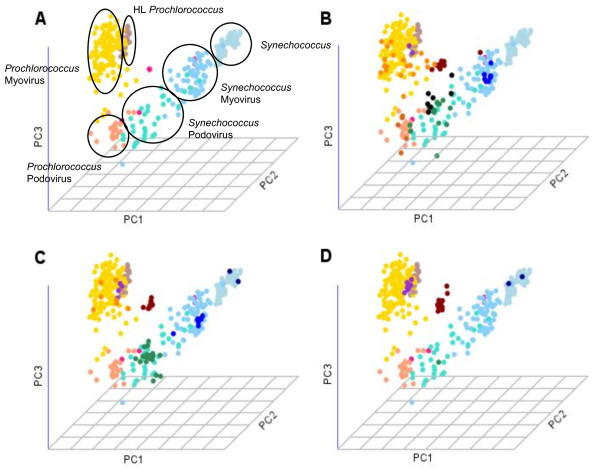
**A. PCA plot based on oligonucleotide frequencies (340 features), projected onto three uncorrelated axes (principal components)**. Each dot represents a psbA sequence color based on GOS classification [11]:*Synechococcus *(light blue), *Synechococcus*-like Myovirus (light sky blue), *Synechococcus*-like Podovirus (turquoise), HL-*Prochlorococcus *(rosy brown), LL-*Prochlorococcus *(deep pink), *Prochlorococcus*-like Myovirus (gold) and *Prochlorococcus*-like Podovirus (light salmon). B. PCA plot showing the distribution of DNA sequences extracted from station M from deep sea in March 2006. The Mediterranean data are presented on the background of the GOS data (GOS sequences are colored as in A.). The Mediterranean data are shown in darker colors *Synechococcus *(royal blue), *Synechococcus*-like Myovirus (blue), *Synechococcus*-like Podovirus (dark green), HL-*Prochlorococcus *(dark purple), LL-*Prochlorococcus *(dark red), *Prochlorococcus*-like Myovirus (dark orange) and *Prochlorococcus*-like Podovirus (dark brown). Black dots represent sequences for which there was no agreement between our independent classifiers. Manual examination suggests that this is a new subclass of sequences not represented in the GOS database. C and D. represent PCA plots showing the distribution of DNA and RNA sequences, respectively. The Mediterranean Sea sequences colored in dark colors (as is B) on the background of the GOS data (colored as in A) were extracted from surface water sampled in March 2006. As demonstrated, the bacterial sequences are distributed similarly between subclasses in both the DNA and RNA sequences, while the viral *psbA*s are mostly found at the DNA level.

To study the distribution of *psbA *genes and transcripts in the marine environment, we sought a method that could further subclassify the fragments into their explicit taxonomic origins: *Synechococcus*, *Synechococcus*-like Myovirus, *Synechococcus*-like Podovirus, high-light adapted *Prochlorococcus *(HL-*Prochlorococcus*), low-light adapted *Prochlorococcus *(LL-*Prochlorococcus*), *Prochlorococcus*-like Myovirus and *Prochlorococcus*-like Podovirus. To date, only a small number of genomes from cultured marine *Synechococcus *and *Prochlorococcus *and their phages are available for training. Specifically, *Synechococcus *podovirus *psbA *sequences and LL-*Prochlorococcus *are underrepresented in culture. To broaden the *psbA *representation, we included in the training set fragments from the GOS expedition that were annotated based on DNA trees (as described in [15]) and on neighboring genes (as described in [12]) [See also Additional File 2]. To first examine whether the *psbA *genes in the training set clustered into distinct groups based on their nucleotide composition, we applied an unsupervised clustering method, Principle Component Analysis (PCA) [44] (see Methods section), to the GOS data representing each sequence by the oligonucleotide frequencies vector. As illustrated in Figure 2A, of the seven groups, six clearly showed distinct clusters (denoted by different colors). The only sequences that did not cluster in the PCA were the LL-*Prochlorococcus *(pink dots), presumably due to their under-representation in the data.

**Figure 3:**
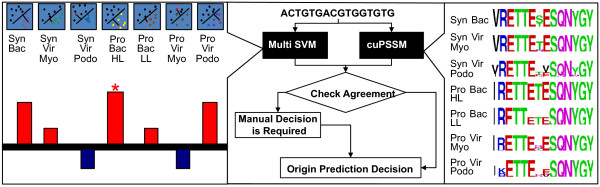
**A summary of the *psb*A sequence classifier**. Each sequence is classified by two independent approaches: multi-class SVM (left) and cuPSSM (right). In the multi-class SVM classifier, each sequence is represented by an oligonucleotide frequency vector (calculated in overlapping windows) and tested against seven different SVM classifiers (trained on culture and environmental data from GOS) [See Additional Files 1 and 2]. The sequence is classified based on the classifier in which it achieved the highest positive result. Independently, the sequence is aligned to a template *psb*A gene and scored against seven different cuPSSMs. The sequence is then classified based on the subgroup for which it achieved the highest score. Finally, the results of the two approaches are compared. Sequences for which the two independent classifiers converged are classified according to the common sub classification. In cases where no agreement exists, the sequence is further classified manually as described in the text.

Although the PCA clustering clearly indicates that *psbA *fragments can be grouped based on their oligonucleotide frequencies, it is not a practical method for the automatic and accurate classification of large amounts of data. Thus, we expanded our binary prediction described above to a multi-class problem. To this end, we built seven new SVM classifiers, one for each taxonomic group. In each classifier, we trained an SVM to separate one of the seven groups from all the other groups using the oligonucleotide frequencies as a feature vector. Subsequently, each fragment was tested on each of the classifiers and was given a discriminate value, denoting the confidence that the fragment belongs to the specific group. Further, the discriminate values produced by each classifier were ranked, and the tested fragment was labeled based on the classifier in which it received the highest positive value. Independently, we built seven cuPSSMs based on the codon usage of the training data. The tested sequences were then aligned to a template *psb*A gene, scored against seven different cuPSSMs (each sequence was trained on a single taxonomic group) and given a label based on the cuPSSM for which it achieved the highest score. To assure an accurate prediction of the testing data, each fragment was examined against the two classifiers independently, and a final label was acquired only when the results of the two classifiers converged. A summary of our classification methodology is illustrated in Figure 3. As illustrated, when given a sequence, it is independently tested by two classifiers: (i) multi-class SVM and (ii) cuPSSM. Further, the results of the two approaches are combined and compared. Sequences for which the two independent classifiers converged are classified according to the common sub-classification (e.g *Prochlorococcus*-like Myovirus). In cases where there is no agreement between the two classifiers (i.e. multi-class SVM vs cuPSSM), sequences are considered "un predicted" and are subjected to a manual decision.

To evaluate the performance of the classifier on an independent dataset, we tested it on a recent dataset of cultured and uncultured viral *psbA *fragments from freshwater and marine environments [35]. As shown in Table S3 [See Additional file 3], there was a 92% agreement for the full-length fragment (59/64) between our predictions (agreed by the two independent classifiers) and the phylogenetic-based annotation [35]. Notably, all 20 sequences originated from culture were predicted correctly by our classifier. Interestingly, the classifier was able to detect two *Synechococcus *podoviruses containing *psbA *genes. These two may be added to the only two currently known cultured *Synechococcus *podovirus [4]. Notably, when applied to the multi-class problem, our method still achieved a relative high performance for a partial length sequence of 400 bp, however, as shown, shorter fragments could not be predicted with high accuracy [See Additional file 4].

We further tested the applicability of the method for the taxonomic classification of the *psbD *gene, which codes for the D2 proteins. In this case, we trained the algorithm on a set of annotated *psbD *genes from culture and tested it on the *psbD *fragments from the GOS. Here again, the prediction of our automatic classifier was compared to independent annotations based on phylogenetics analysis[15] and neighboring genes[12] [details annotation is shown in Table S5, Additional file 5]. As shown in Table S5, out of the 530 annotated sequences, we correctly predicted 515 (97%). Of the 15 missed annotations, in 13 the method did not converge (nine *Prochlorococcus *Myovirus and four *Synechococcus *Myovirus), while only two *Synechococcus *bacterial sequences were predicted incorrectly as *Synechococcus *viral sequences.

### Studying psbA dynamics in the Mediterranean Sea based on supervised classification

Confident with the test results on *psbA *and *psbD *gene fragments, we applied our classifier to DNA sequences from the Mediterranean Sea. The prediction results for the sequences extracted from all stations at different times of the year are summarized in Table 3. As shown, in 867 of the 894 *psbA *fragments (97%), the classifications to one of seven taxonomic groups based on the SVM and the cuPSSM were identical. For validation, we again applied a PCA analysis to the test data from the Mediterranean and superimposed it on the fully informed training data (fixing the principle components as described in the Methods section). Figure 2B illustrates the test results for station Tb01 from the Mediterranean Sea (described in detail below) plotted on the background of the GOS training data. As shown, samples from the test data in the majority of cases (highlighted in dark colors) fell within the corresponding clusters from the training set, i.e., sequences from the training set that were classified by our prediction method as being *Synechococcus*-like podo viruses (dark green) fell within the cluster of the same subclass from the GOS data. Interestingly, the seven sequences for which there was no classification agreement in our classifier (black dots) did not coincide with a specific cluster, and rather appeared to be located between the bacterial and viral branches.

**Table 3:** Summary of prediction results for *psbA *DNA and RNA sequences extracted from the Mediterranean Sea.

	**Loc**^#^	**Date**	**Depth**	**Seq**^$^	**PH**	**PL**	**S**	**PM**	**PP**	**SM**	**SP**	**N**^&^	T*
A	Tb01	Jan 07	surface	DNA	1	14	6	11	6	2	8	2	50

B	Tb01	Jan 07	surface	RNA	7	18	0	0	0	0	0	0	25

C	Tb04	Jan 07	surface	DNA	2	7	0	12	7	3	46	0	77

D	Tb04	Jan 07	surface	RNA	2	4	0	0	0	0	0	0	6

E	Tb01	Oct 06	surface	DNA	1	1	50	7	0	1	6	0	66

F	Tb01	Oct 06	surface	RNA	4	4	8	0	0	0	0	0	16

G	Tb01	Oct 06	DCM	DNA	0	11	0	48	14	0	9	6	88

H	Tb01	Oct 06	DCM	RNA	1	45	0	0	0	0	2	0	48

I	Tb04	Oct 06	surface	DNA	0	0	3	0	0	10	51	2	66

J	Tb04	Oct 06	surface	RNA	0	0	10	0	0	0	0	0	10

K	Tb01	Mar 06	surface	DNA	4	1	7	18	3	8	14	1	56

L	Tb01	Mar 06	surface	RNA	6	2	7	0	0	0	0	0	15

M	Tb01	Mar 06	DCM	DNA	2	13	0	19	6	8	15	7	70

N	Tb01	Mar 06	DCM	RNA	6	51	7	0	0	0	0	0	64

O	Tb04	Mar 06	surface	DNA	4	11	2	7	0	8	42	0	74

P	Tb04	Mar 06	surface	RNA	9	17	2	0	0	0	1	0	29

Q	Tb01	May 06	surface	DNA	0	0	11	3	0	14	11	1	40

R	Tb01	May 06	surface	RNA	10	0	3	0	0	0	0	0	13

S	Tb01	May 06	DCM	DNA	0	17	0	4	0	0	3	9	33

T	Tb01	May 06	DCM	RNA	1	48	0	0	0	0	0	0	49

					**60**	**264**	**116**	**129**	**36**	**54**	**208**	**28**	**895**

^# ^– Location, Station; ^$ ^– Sequence; ^&^– No decision; * – Total PH – *Prochlorococcus *HL- Bacteria; PL – *Prochlorococcus *LL- Bacteria; S – *Synechococcus *Bacteria; PM – *Prochlorococcus *Myovirus; PP – *Prochlorococcus *Podovirus; SM – *Synechococcus *Myovirus; SP – *Synechococcus *Podovirus

### Estimation of sampling efficiency

**Figure 4:**
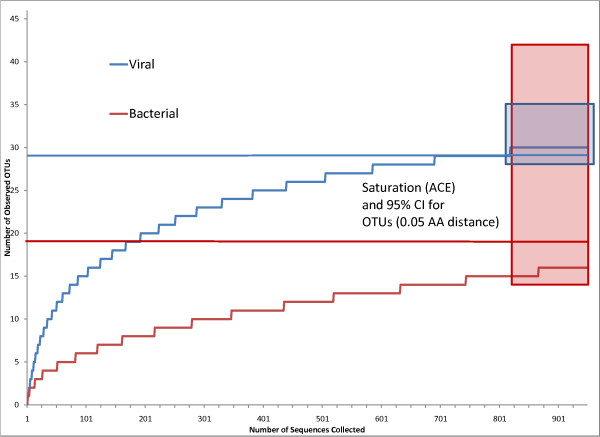
**Rarefaction analysis of bacterial and viral *PsbA***. Median rarefaction curve for 1,000 replicates, sampling without replacement from observed OTUs (0.05 amino acid distance); rectangles and horizontal lines indicate the 95% confidence interval and expected value for the actual number of OTUs in the sampled population (ACE).

In order to evaluate if our PCR samplings approximated the natural *psbA *diversity in our samples, we performed a naïve rarefaction analysis of both the RNA and DNA sequences. The analysis suggested that each sampling was performed more thoroughly on the DNA population than on the RNA sequences, yet both were sampled very thoroughly. Sampling of translated sequences was saturated (95%) at distances of 0.09 for the RNA samples (61% at 0.02) and 0.02 for the DNA samples. Separating the collection not by the collection method (from RNA or DNA) but instead into pools of predicted bacterial and viral origins (Fig. 4) demonstrates that the bacterial sampling is saturated at an amino acid distance of 0.10 and the viral at 0.05.

### psbA dynamics at the DNA level

**Figure 5:**
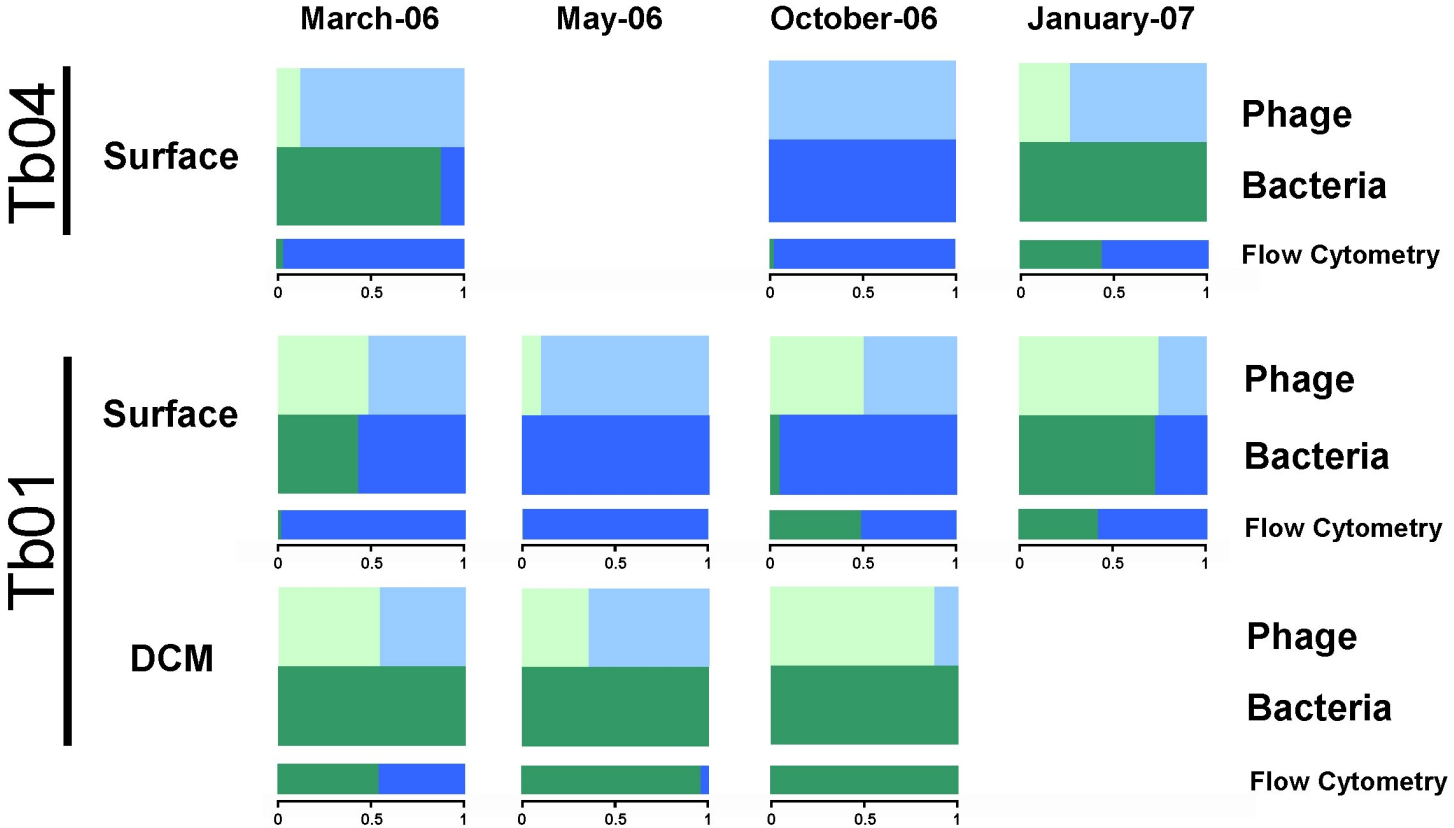
**A summary of the relative frequency of cyanobacteria and phage *psbA *sequences at the DNA level as predicted by our classifier**. Flow cytometry results are given in comparison. Host and phage sequences are divided into four groups:*Synechococcus *(blue); *Prochlorococcus *(green); *Prochlorococcus*-like phage (light green); and *Synechococcus*-like phage (light blue).

Based on our predictions, we found that the pelagic station (Tb01) DCM (Deep Chlorophyll Maximum) depth (at all sampling dates) was dominated by one bacterial group (LL-*Prochlorococcus*), while viral sequences belonged to two groups (i.e., *Prochlorococcus*- and *Synechococcus*-like) (Fig. 5 and Table 3). A similar trend was observed at the surface water of station Tb04 in January 2007, with *Synechococcus*-like phages and only *Prochlorococcus*-like hosts (Table 3). In addition, in surface water at station Tb01 in May 2006, *Prochlorococcus*-like phages sequences were detected, while no *Prochlorococcus-*like hosts were observed. Our observations from station Tb01 (DCM depth in October 2006 and from the surface in May 2006) were also confirmed when compared to flow cytometry results applied to the same samples. These results again indicated that only one bacterial group is present while the phages carry *psbA *genes from both bacterial groups. Usually, cyanobacterial host genes tend to be acquired by host-like phages (i.e., *Synechococcus *genes by *Synechococcus *phages and *Prochlorococcus *genes by *Prochlorococcus *phages) [16]. However, rare events in which phage *psbA *did not cluster with their hosts and did not have *psbA *isoforms consistent with that of their hosts were also observed (PCR based observations reported by [16]). This phenomenon was hypothesized to be the result of gene swapping when two or more different viruses infect the same host [16]. Our observations further support the hypothesis that these swapping events occur when different viruses (*Prochlorococcus*-like or *Synechococcus*-like phages) infect the same host [45].

### psbA dynamics at the RNA level

Among all RNA-derived clones, only a single *psbA *sequence was classified as a *Synechococcus*-like podovirus while an additional seven sequences (sample D1-H collected from station Tb01) gave conflicting scores (see Table 3). These sequences had very low identity (~85% at the nucleic acid level) to *Prochlorococcus*. In order to examine these sequences, we tested several features: (1) *psbA *GC content; (2) D1 protein signature motif; (3) PCA topologies; and (4) *psbA *tree topologies. A simple analysis of their nucleotide contents showed that the GC content is 47–48%, which is consistent with the GC content of *Synechococcus*-like phages (47–50%) (while the calculated GC content of *Synechococcus *and *Prochlorococcus *and their phages was 58–60% and 44–45%, respectively [15]). When applying a phylogenic analysis, all seven sequences were clustered with *Synechococcus*-like podoviruses, but had a common branch with *Prochlorococcus *MIT9211 in the *psbA *tree (data not shown). Taken together, we suspect that the seven clones are *Synechococcus*-like phages. However, the deposition of additional future *psbA *sequences in the GenBank could modify our predictions and classify these sequences into another group.

### psbA dynamics at both RNA and DNA levels

When comparing *psbAs *genes and transcripts, we observed that RNA and DNA levels correlated for different predicted bacterial groups, whereas no such correlation was observed in phages (Table 3). Figure 2 illustrates the distribution of DNA (Fig. 2C) and RNA (Fig. 2D) samples among the seven subclasses in station Tb04 from surface water (sample D1-O (DNA) and D1-P (RNA)) plotted on the background of the clusters from the GOS training data. As shown, while *Synechococcus*-like podoviruses (green) are most abundant in the DNA samples, only one was predicted in the RNA samples. Nevertheless, the bacteria sequences were distributed similarly in the RNA and DNA samples from the same station. Interestingly, in both DNA and RNA samples, we observe a relatively large cluster of LL-*Prochlorococcus *sequences (dark brown). As discussed above, the latter group was underrepresented in the training data and did not generate a distinct cluster. Other cases where host genes were predicted only in the RNA could be explained by a high expression of these *psbA *genes, along with a low cell abundance. The opposite phenomenon (i.e., host genes predicted only in DNA) could possibly result from low expression of *psbA *at the time of sampling. Overall, 73% of the DNA-derived clones were classified as cyanophages, whereas less than 1.5% of the RNA-derived clones were classified as phages.

### D1 viral/bacterial motif search

Amino acid variations in the variable loop between helices D and E (R/K ETTXXXSQ/H) were observed recently in the viral fraction of the GOS data [12]. This D1 PEST-like domain is implicated as the site of initial cleavage in the D1 protein and is assumed to be important for D1 turnover rate. As our classification method does not rely on this variable region, we were interested in examining this phenomenon in the Mediterranean Sea data. As previously observed, D1s assigned to bacteria mainly contained the known ESE and EAE cyanobacterial motifs while the viral sequences contained 18 different motif variants [See Additional file 6], most of which were the same viral motifs previously observed in the GOS data [12]. Several different new viral motifs were observed only in the Mediterranean samples (EKE, ESV, DIE, DTE and GLI). Interestingly, one viral motif (observed on both the RNA and DNA levels) completely deviated from the canonical R/KETTXXXSQ/H cyanobacterial motif and included changes throughout the entire loop (RETS EKESL; see Additional file 7, amino acid alignment plus schematics). It is important to note that our classifier not only does not rely on loop sequence, but achieves identical classifications when these sequences are removed from the data. To better understand the nature of these unique D1s, we searched the database for similar *psbA *sequences. One GOS sequence (JCVI_SCAF_1096628349996) had a 100% match to RNA D1 clone P_E8 and also included two predicted hypothetical cyanophage ORFs (See Additional file 7), further confirming our viral affiliation predictions.

## Conclusion

In this paper we employed two independent classification methods for identifying the origin of *psbA *genes: 1) cuPSSM, based on position specific codon usage; and 2) a machine learning approach (SVM) trained on oligonucleotide (specifically tetranucleotide) composition. Combining the two approaches, we built a classifier for rapid and accurate annotation of core photosystem-II genes and transcripts to seven taxonomic classes of cyanobacteria and cyanophages. When tested on independent datasets from culture and from the environment, the method demonstrated a very high accuracy, ranging from 92–100% true predictions for *psbA *and *psbD *gene fragments. When tested specifically on short sequences, we showed that for binary classification, the method can accurately predict very short partial gene fragments (down to 100 bp) while the multi-class classifier achieved a high performance for partial fragments of 400 bp and longer. The great advantage of this classification method is that it does not rely on having the whole gene and can be applied to partial sequences that could be derived in the future from rapid sequencing methods without the need for assembly processes. However, as a supervised approach, accurate classification requires the availability of reliable annotated training data for the gene of interest, and cannot be applied for annotating any random sequence extracted from the environment.

Finally, we applied the method to rapidly classified *psbA *DNA and RNA sequences extracted from the Mediterranean Sea, and studied the spatial distribution of the host and viral genes and transcripts from different depths and at different seasons. Our results show that bacterial *psbA *gene and transcript levels were highly correlated, whereas no such correlation was observed in phages, which were observed mainly on the gene level and rarely as transcripts.

## Methods

### Sample collection

Seawater samples were collected during four cruises (March, May and October 2006 and January 2007) on board the R/V Mediterranean Explorer. Two locations were sampled: near-shore station Tb04 and open sea station Tb01. Station Tb04 is located 20 km off the coast (32°09'N, 34°34'E) at ca. 200 m bottom depth, and station Tb01 is located 51 km offshore (34°14'E, 32°10'N) at ca. 1,000 m bottom depth. Surface water samples were collected from both stations, while samples from the DCM were collected only from station Tb01. Twenty liters were pre-filtered through a GF/A glass-fiber filter (Whatman) or a 3 μm polycarbonate filter (GE Water & Process Technologies); the plankton in the filtrate was collected on a 0.2 μm Sterivex filter (Milipore, MA, USA) using a peristaltic pump (Cole Parmer Masterflex 5, channel hardware). After collection, the Sterivex filters were filled with 1 ml of lysis buffer [46] and stored at -80°C.

### Nucleic acid extraction

Nucleic acid extraction was performed according to Massena *et al*. [46] with several modifications. Nucleic acid extraction began with the addition of 20 μl of 100 mg/ml fresh lysozyme (final concentration 2 mg/ml) to the Sterivex filter and incubation with rotation at 37°C for 30 min. One hundred μl of 10 mg/ml Proteinase K (final concentration 1 mg/ml) and 50 μl of 20% (w/v) sodium dodecyl sulfate (SDS) (final concentration 0.5% (w/v)) were added and the filter was incubated at 55°C for 1 h. The lysate was then recovered from the filter and extracted with an equal volume of phenol pH 8.0. The aqueous phase was extracted with an equal volume of phenol-chloroform-isoamyl alcohol (25:24:1; pH 8.0), then extracted with an equal volume of chloroform-isoamyl alcohol (24:1). One tenth volume of 3 M NaOAc and 2 volumes of absolute ethanol were added to the aqueous extract. The extracts were kept overnight at -20°C. Following 20 min centrifugation at maximal speed at 4°C, the pellet was washed with 1 ml ice-cold 80% ethanol and centrifuged for 10 min at maximal speed at 4°C. The pellet was air-dried and resuspended in 40 μl ultra pure water. Half of the final extract (20 μl) was stored as genomic DNA at -20°C. The RNA was treated with RNase-Free DNase I (Ambion, Cambridgeshire, UK) for 30 min at 37°C to remove DNA contamination. DNase was inactivated by heat denaturation at 75°C for 10 min and samples were stored at -80°C for further use. DNA and RNA concentrations were determined by measuring their UV absorbance and UV_260_:UV_280 _ratios.

### Reverse transcription and PCR amplification

Total RNA (100–300 ng) was reverse transcribed with *psbA *degenerate reverse primer psbA-2R from [4] using Bio-RT (Bio Lab) according to the manufacturer's instructions. Reaction mixtures were incubated at 37°C for 1 h. *PsbA *gene fragments (~750 bp) were amplified by PCR from cDNA and genomic DNA using the degenerated PCR primers designed by [4] that target the conserved YPIWEA and HNFPLD regions. PCR was performed in a total volume of 25 μl containing 10 ng of template DNA/cDNA, 2.5 μl of 10 × OptiBuffer, 2 μl of dNTP, 1.3 μl of 50 mM MgCl_2_, 1 μl of 25 μM psbA-1F (TAYCCNATYTGGGAAGC), 1 μl of 25 μM psbA-2R (TCRAGDGGGAARTTRTG) and 1.2 U of BIO-X-ACT™ (Bioline, London, UK). The amplification conditions comprised steps at 95°C for 2 min, 30 cycles at 94°C for 1 min, 55°C for 1 min, and 68°C for 1 min followed by one step of 7 min at 68°C. We performed two tests for the presence of contaminating DNA in the RNA sample: (1) PCR on the RNA samples without the reverse transcription step; and (2) treating the RNA samples with RNase and subjecting them to RT-PCR. To test the reagents for DNA contamination, PCR reactions without template were performed.

### Cloning of psbA genes, library construction and sequencing

PCR products were cloned using the QIAGEN PCR cloning kit according to the manufacturer's instructions. Clones were picked randomly into 20 96-well plates. Each 96-well plate represented different dates (March, May and October 2006 and January 2007), stations and depths (Tb04 surface, Tb01 surface and Tb01 DCM), and sources (DNA and RNA). All plates were sequenced at the MPI for Molecular Genetics in Berlin. Sequences were deposited in GenBank under accession numbers EU727548–EU728419.

### Flow cytometry

Samples of 1.8 ml were taken directly from the Niskin bottles and fixed immediately at room temperature with 140 μl of 25% glutaraldehyde (sigma G-5882) for 20 min, after which they were frozen in liquid nitrogen. In the laboratory, the samples were kept at -80°C until further analysis was made. Samples were thawed at 37°C and analyzed by excitation using an argon laser (488 nm) for either 10–15 min or until 10,000 cells were counted. Taxonomic discrimination was based on the following parameters: cell side scatter – a proxy of cell volume; forward scatter – a proxy of cell size; and pigment identification via orange and red fluorescence for phycoerythrin and chlorophyll (585 nm and 630 nm, respectively). Beads (0.93 μm Polysciences™) served as a standard.

### Position-Specific Scoring Matrix (PSSM/cuPSSM)

PSSM calculates and demonstrates the variation found in each position of the multiple sequence alignment [47]. In PSSM and cuPSSM, each row entry corresponds to a different amino acid in the D1 protein or to a different codon in the *psb*A gene, respectively. All sequences tested were aligned based on the protein sequence and reversed to DNA for the cuPSSM using MUSCLE protein multiple alignment software [48]. Odd scores Oi(n) for PSSM were calculated from the raw counts in the PSPM. Odd scores were defined as Pi(n)/P(n), where Pi(n) is the probability of amino acid/codon n at position I, and P(n) is the background frequency.

### Support Vector Machine (SVM)

SVM experiments were carried out with Gist Program version 2.1.1. [49]. Input data were normalized by rescaling the columns to values between -1 and 1. A linear kernel was applied for all SVM classifiers. Each sequence was represented by an input feature vector. The features included normalized frequencies of: 1) mononucleotides (i.e., A, T, G, C); 2) dinucleotides (16 pairs); 3) trinucleotides (64 triplets); and 4) tetranucleotides (256). In all cases, frequencies were calculated from both DNA strands (coding and non-coding) in overlapping windows and normalized for the sequence length. To evaluate SVM performance, a ROC (Receiver Operating Characteristic) curve describing the relationship between the False Positive Rate (FPR) and the True Positive Rate (TPR) were plotted. The Area Under the Curve (AUC) was reported for each SVM test.

For statistical analysis, we calculated the percent of correctly predicted sequences (accuracy), as well as the sensitivity and specificity for each test based on the trusted GOS labeling [11]:







### Multi-class SVM

The multi-class SVM approach, also called the One Versus All approach, is generally a series of binary SVM classifiers, whereby the members of one subclass (one) in each classifier are separated from the rest of the data (all) [50]. For a given query, the predicted subclass is defined according to the classifier for which it achieved the highest positive discriminating value. In the current study, we built seven subclasses, one for each taxonomic group.

### Principal Components Analysis (PCA)

The PCA figures represented in this work were produced in a two-step procedure. First, standard PCA was performed for the training set, including only culture and GOS data, using oligonucleotide frequency vectors (340 features). Second, coordinates for the testing set were calculated with respect to the first three principal components derived from the first step. Color labeling for the testing set was based on prediction agreement (by Multi-class SVM and cuPSSM methods). In case of prediction disagreement, points were colored black. The PCA was conducted using GNU R software [51].

### Rarefaction analysis and community structure analysis

Aligned DNA sequences (878 *psb*A sequences), which had been predicted to be viral or bacterial in origin, were used to infer a phylogeny with PhyML [52]. The data were analyzed as DNA sequences and translated again for each analysis. The resulting trees were broken down into a distance matrix (using MATLAB); these distances formed the basis for a rarefaction analysis with DOTUR [53]. The sequences were analyzed as an entire group as well as in subsets: RNA/DNA derived sequences and viral/bacterial sequences.

## Availability

A standalone package called MgFC (MetaGenomic Fragment Classification) suitable for linux OS is available for download from 

## Abbreviations

GOS: Global Ocean Sampling; HGT: Horizontal Gene Transfer; SVM: Support Vector Machine; cuPSSM: Codon Usage Position Specific Scoring Matrix; PCA: Principle Component Analysis.

## Authors' contributions

ST participated in the development of the computational methodology, performed the predictions and statistical analysis and drafted the manuscript. DM participated in the design of the study, carried out the experiments and drafted the manuscript. BCK carried out the rarefaction analysis. TY and IBF analyzed the flow cytometry results. MFP coordinated the rarefaction analysis. OB conceived the study, and participated in its design and coordination. YMG conceived and coordinated the study, designed the computational methodology. All authors read and approved the final manuscript.

## Supplementary Material

Additional file 1 ***Synechococcus *host and phage sequences from cultured data**. The data provides the sequences from cultured data used for training the algorithm. Click here for file

Additional file 2 ***Synechococcus *and *Prochlorococcus *host and phage sequences from environmental data**. The data provides the sequences from environmental data used for testing the algorithm. Click here for file

Additional file 3 **Detailed prediction results on and independent dataset from culture and Marine environment**. The data provides the detailed result of the predictions using MgFC algorithm compared to phylogenetic-based annotation [35]. Click here for file

Additional file 4 **Summary of prediction results on and independent dataset from culture and Marine environment**. The data provides the detailed result of the predictions using MgFC algorithm compared to phylogenetic-based annotation [35]. Click here for file

Additional file 5 **Prediction results for *psb*D fragments**. The data provides the summary of the prediction results for *psb*D using MgFC. Click here for file

Additional file 6 **Occurrence of different D1 R/KETTXXXSQ/H motifs in viral and bacterial assigned Mediterranean Sea D1 sequences**. Asterisks denote statistically significant motifs (*p*-value < 0.01), *p*-values were calculated using the hypergeometric distribution test, applying the Bonferroni correction for multiple testing. Click here for file

Additional file 7 **Schematic representation and alignment of GOS scaffolds containing the new viral motif EKE**. Red arrows represent viral predicted ORFs (#1 denotes hypothetical protein p158 from cyanophage S-PM2, and #2 denotes unknown protein from cyanophage P60), gray arrow represents an unknown ORF, and blue arrow represents the D1 protein. Click here for file
