# Supplementary material for: A supervised learning approach for taxonomic classification of core-photosystem-II genes and transcripts in the marine environment
Source: BMC Genomics. 2009 May 16;10:229. doi: 10.1186/1471-2164-10-229 (PMC2696472; doi:10.1186/1471-2164-10-229)
Supplement: Additional file 4 — Summary of prediction results on and independent dataset from culture and Marine environment. The data provides the detailed result of the predictions using MgFC algorithm compared to phylogenetic-based annotation [35]. [file 1471-2164-10-229-S4.pdf]

Table S4: Summary of the MgFC classifier results tested on an independent data from [1], using partial fragments

Sequence Length	Number of runs	Fraction of sequences with confirmed results*	Standard deviation
400	*25*	*0.85*	*0.03*
300	*30*	*0.75*	*0.04*
200	*50*	*0.72*	*0.04*
100	*100*	*0.63*	*0.05*

* Sequences were considered confirmed only when both our classifiers converged and the prediction results fitted the original prediction.

Reference

1. Chenard C, Suttle CA: **Phylogenetic diversity of sequences of cyanophage photosynthetic gene psbA in marine and freshwaters**. *Appl Environ Microbiol* 2008, **74**(17):5317-5324.
