# Supplementary figures and images for: A supervised learning approach for taxonomic classification of core-photosystem-II genes and transcripts in the marine environment
Source: BMC Genomics. 2009 May 16;10:229. doi: 10.1186/1471-2164-10-229 (PMC2696472; doi:10.1186/1471-2164-10-229)

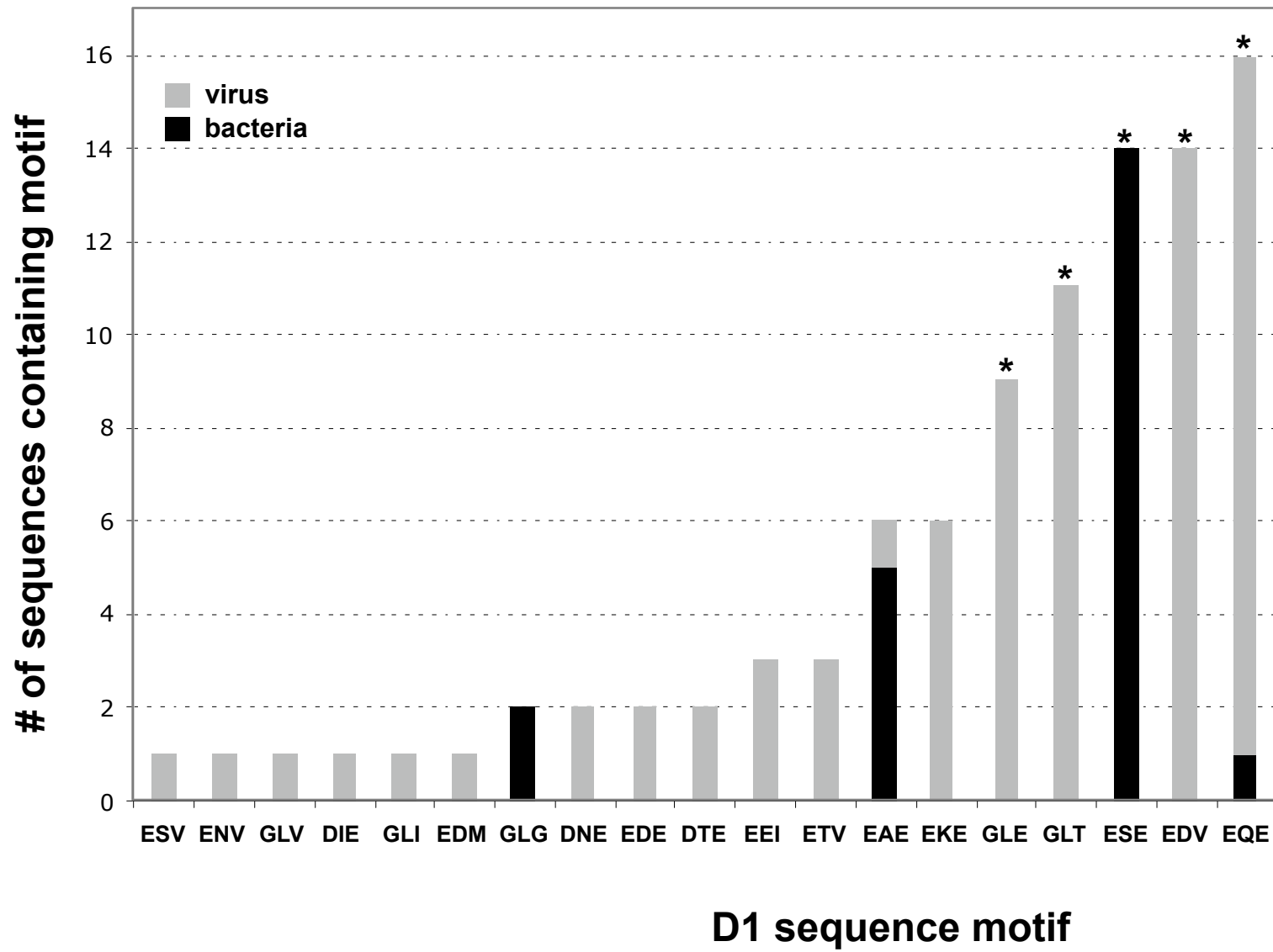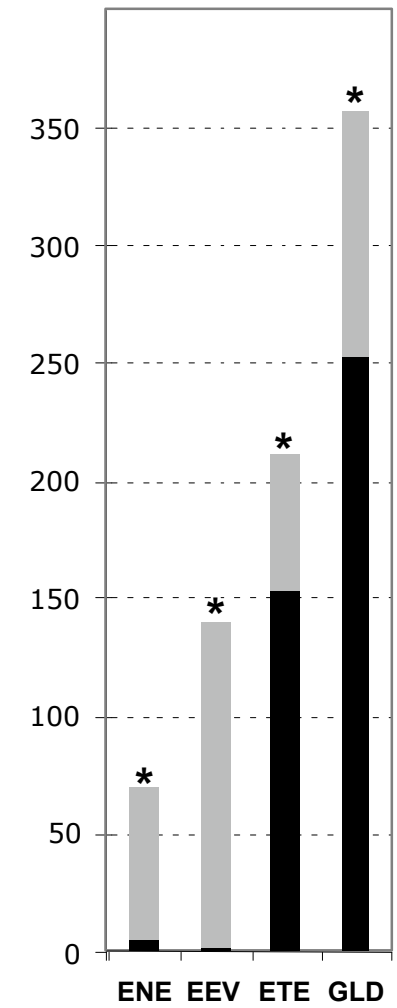

Supplement: Additional file 6 — Occurrence of different D1 R/KETTXXXSQ/H motifs in viral and bacterial assigned Mediterranean Sea D1 sequences. Asterisks denote statistically significant motifs (p-value < 0.01), p-values were calculated using the hypergeometric distribution test, applying the Bonferroni correction for multiple testing. [file 1471-2164-10-229-S6.pdf]
