# Supplementary material for: A supervised learning approach for taxonomic classification of core-photosystem-II genes and transcripts in the marine environment
Source: BMC Genomics. 2009 May 16;10:229. doi: 10.1186/1471-2164-10-229 (PMC2696472; doi:10.1186/1471-2164-10-229)
Supplement: Additional file 7 — Schematic representation and alignment of GOS scaffolds containing the new viral motif EKE. Red arrows represent viral predicted ORFs (#1 denotes hypothetical protein p158 from cyanophage S-PM2, and #2 denotes unknown protein from cyanophage P60), gray arrow represents an unknown ORF, and blue arrow represents the D1 protein. [file 1471-2164-10-229-S7.pdf]

1096628349996

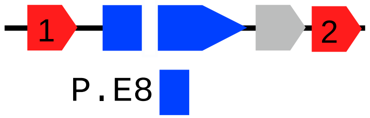

P.E8

1096628349996	ETSEKESLNNGYKFGQQEE
**P.E8**	**SAMHGSLVTSSLVRETSEKESLNNGYKFGQQEE**
EN138731	**SAMHGSLVTSSLVRETTEKESLNYGYKFGQEEE**
